# Association of dietary niacin intake with diabetic retinopathy: A cross-sectional study

**DOI:** 10.1097/MD.0000000000046064

**Published:** 2025-11-21

**Authors:** Shilong Wang, Xiao Li, Meirong Chen

**Affiliations:** a First Clinical Medical College, Shandong University of Chinese Medicine, Jinan, China; b First Clinical College of Chinese Medicine, Hunan University of Chinese Medicine, Changsha, China; c Department of Ophthalmology, Shandong Hospital of Traditional Chinese Medicine, Jinan, Shandong Province, China.

**Keywords:** cross-sectional study, diabetic retinopathy, dietary niacin, NHANES, nutrition

## Abstract

This study explores the association between dietary niacin and diabetic retinopathy (DR). Data from the National Health and Nutrition Examination Survey (NHANES) 2005–2018 were used in this cross-sectional investigation. The nonlinear relationship between dietary niacin and DR was investigated using weighted multivariate logistic regression and generalized additive models. The threshold effects were also calculated using a 2-stage linear regression model. In addition, subgroup analyses incorporating interaction assessments were undertaken. For this study, a total of 3990 eligible participants were included. DR had a negative association with dietary niacin after accounting for all variables (OR = 0.92, 95% CI: 0.86, 1.00, *P* = .044), and this association remained stable across all subgroups. A nonlinear association was found in male subjects with an inflection point of 3.35 (10 mg). The discovery of a negative relationship between dietary niacin and DR in this research provides empirical support for the development of more sane dietary guidelines. It also suggests that daily dietary management enriched with niacin may be a feasible strategy with economic effects to improve DR.

## 1. Introduction

As the prevalence of diabetes continues to rise, so do the public health problems caused by diabetes complications.^[1]^ Diabetic retinopathy (DR) stands out as a highly consequential microvascular complication arising from diabetes, serving as a leading cause of blindness.^[2,3]^ According to studies, almost 1 in 9 of the 246 million diabetics globally have retinopathy that poses a risk to their vision.^[4]^ In addition to affecting eyesight, DR increases the risk of systemic vascular disease, increasing the financial strain on healthcare.^[5–7]^ Niacin, recognized by its alternative nomenclature of “vitamin B3” or “vitamin PP,” instigates the generation of biologically efficacious coenzymes, namely nicotinamide adenine dinucleotide (NAD) and nicotinamide adenine dinucleotide phosphate (NADP).^[8]^ Both play crucial roles in redox processes.^[9]^ Prior research has concomitantly ascertained the capacity of niacin to ameliorate the functional integrity of endothelial cells as well as modulate the intricate process of lipid metabolism.^[10,11]^ Numerous antecedent investigations have undertaken a comprehensive assessment of the intricate nexus between niacin and diabetes mellitus. However, these studies have primarily addressed the effects of niacin supplementation and lacked an assessment of daily dietary niacin intake.^[12,13]^ Currently, there is scant knowledge about the correlation between niacin consumption and diabetic retinopathy in the general population. This study aims to fill the knowledge gap and provide empirical support for more rational public nutrition policies.

## 2. Materials and methods

### 2.1. Study population

**Figure 1.**
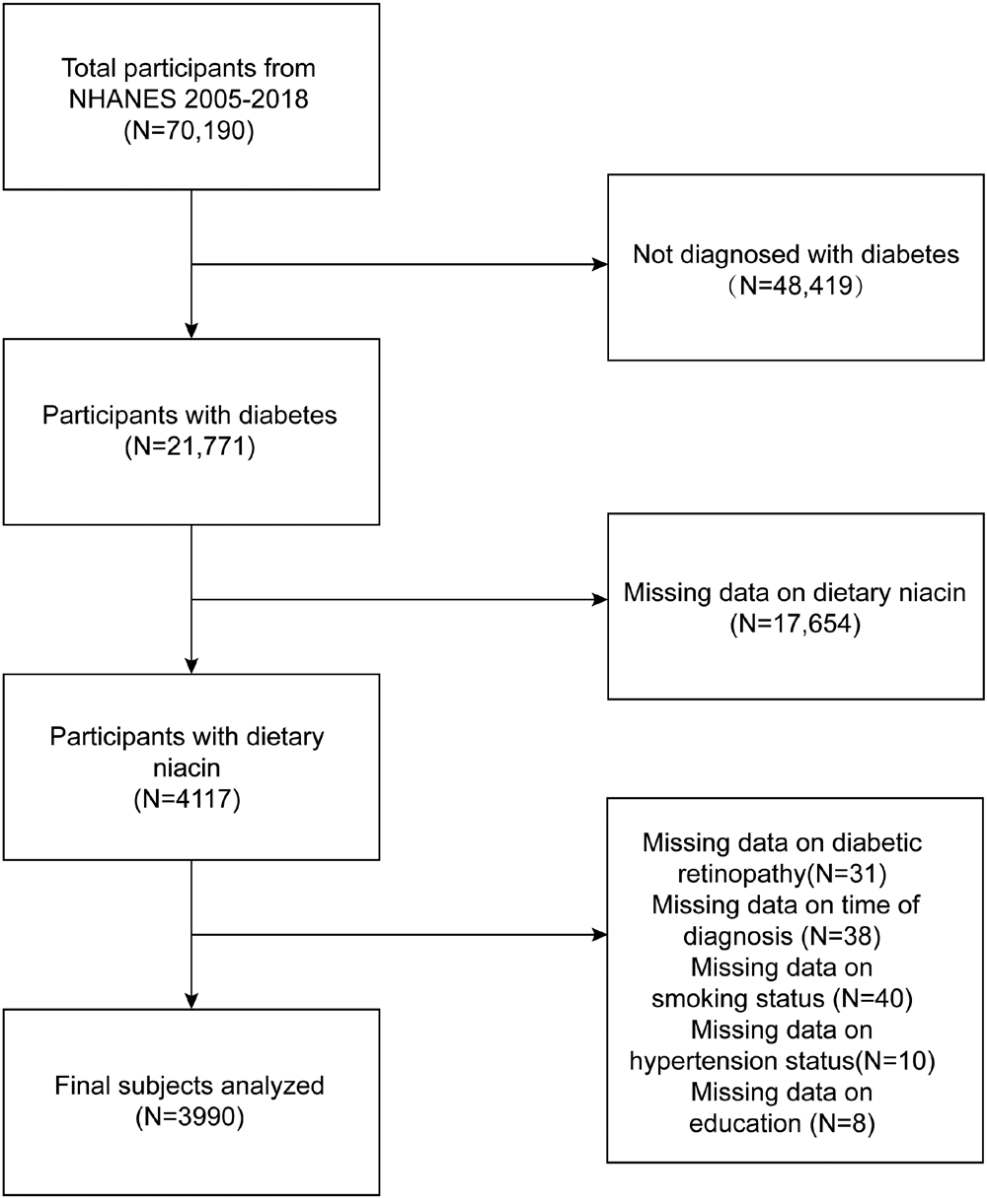
Flow chart of participants selection.

NHANES is the basis for a cross-sectional study designed to determine the health status of U.S. citizens through the collection of objective human health data to identify the healthcare needs of the population and prevent future health problems.^[14,15]^ A complex multistage stratified probability sampling design was employed to sample the U.S. noninstitutional civilian population, with oversampling applied to specific demographic subgroups such as ethnic minorities and the elderly. Corresponding sampling weights were applied in the analysis to account for survey design factors while ensuring the specific representativeness of the subjects.The study combines interviews and physical examination and is administered by the National Centre for Health Statistics (NCHS). Data from the study will be used in epidemiological studies and health science research to help expand the nation’s health knowledge and develop more robust public health policies. The Research Ethics Review Board of the NCHS gave its approval to the protocol of the NHANES study, with all the participants providing their written informed consent. The complete details regarding the NHANES procedure and data can be accessed at https://www.cdc.gov/nchs/nhanes/available. The total number of participants in this cross-sectional study was 70,190. Of these, 48,419 were not diagnosed with diabetes. Seventeen thousand six hundred fifty-four were missing data on dietary niacin, 31 were missing data on diabetic retinopathy, 38 were missing data on the time of diagnosis of diabetes, 40 were missing data on smoking status, 10 were missing data on blood pressure status, and 8 were missing data on education, resulting in a final total of 3990 participants included in this study (Fig. 1).

### 2.2. Ascertainment of DR

Those who answered “yes” to the question “Have you been told by your doctor that your diabetes affects your eyes?” in the questionnaire were defined as having diabetic retinopathy.

### 2.3. Ascertainment of dietary niacin

The dietary interview portion was titled “What We Eat in America.” Each participant was granted the opportunity to engage in 2 24-hour dietary recall interviews. The initial dietary recall interview takes place face-to-face at the Mobile Examination Centre. Three to ten days later, the second interview is taken over the phone. All dietary investigators were required to be trained and receive hands-on interview training before working independently. The dietary information regarding our eating habits in the United States was gathered through the utilization of the Automated Multiple Pass Method, a data collection tool developed by the U.S. Department of Agriculture. The U.S. Department of Agriculture has developed a computer-assisted food coding and data management system called Survey Net, into which all interview data were imported. To ensure data quality, various types of assessments were carried out after the coding of the intake data. Visit https://wwwn.cdc.gov/nchs/nhanes/continuousnhanes/default.aspx?BeginYear=2007 for further details on the dietary interview component and related survey methodologies.

### 2.4. Covariates

Gender, age, race, poverty-to-income ratio, level of education, lifetime use of at least 100 cigarettes, BMI, triglycerides, total cholesterol, HDL, LDL, duration of diabetes, and the status of hypertension were all covariates in the study. Those who responded “yes” to the question, “Were you told by a doctor or other health professional that you had hypertension, also called high blood pressure? “referred to as hypertension. Duration of diabetes was calculated by subtracting the patient’s current age from the age at which the patient was first diagnosed with diabetes. The public may get full measurement methods for each research covariate at www.cdc.gov/nchs/nhanes/.

### 2.5. Statistical analyses

After categorizing the participants based on their DR status, we assessed the disparities among the subjects by employing weighted chi-square tests for categorical variables and weighted Student *t* tests for continuous variables. Categorical variables were described using proportions, while continuous variables were represented by averages and standard deviations. The association between dietary niacin and DR in various models was examined using multiple logistic regression analysis. Model 1 failed to account for covariates, whereas Model 2 made adjustments for certain covariates, and Model 3 accounted for all covariates. Dietary niacin was converted from a continuous variable to a categorical variable (quartiles) for sensitivity analysis. To conduct a sensitivity analysis, we categorized dietary niacin, previously represented as a continuous variable, into quartiles. Smooth curve fitting and generalized additive modeling were applied to examine nonlinearity. A 2-stage linear regression model was employed if nonlinear correlations were seen to fit each interval and calculate threshold effects, which were then checked by a log-likelihood ratio test to determine if a threshold existed. Subgroup analyses by sex, age, and smoking status were also performed and interactions were verified. Missing values were entered from the median of continuous variables. The statistical significance level was established at a cutoff point of *P* < .05. We executed all statistical analyses by employing R (http://www.r-project.org) and EmpowerStats (http://www.empowerstats.com).

## 3. Results

### 3.1. Baseline characteristics

In this cross-sectional study, a total of 3990 participants who satisfied the criteria were included. Compared to the non-DR group, subjects in the DR group had lower income–poverty ratios, were more likely to be hypertensive, with longer duration of diabetes, and had lower dietary niacin intake (*P* < .05). Baseline characteristics of the subjects are displayed in Table 1.

**Table 1:** Characteristics of participants grouped with or without DR.

	Non-DR	DR	*P*-value
	N = 3158	N = 832	
Age (yr)	61.75 ± 12.94	62.39 ± 12.37	.316
Gender (%)			.139
Male	50.60	53.49	NA
Female	49.40	46.51	NA
Race (%)			.235
Non-Hispanic White	37.33	33.77	NA
Non-Hispanic Black	27.55	30.05	NA
Mexican American	17.45	16.83	NA
Other Hispanic	9.50	9.74	NA
Other	8.17	9.62	NA
Education (%)			.167
Less than high school	32.24	34.74	NA
High school	23.53	24.64	NA
More than high school	44.24	40.62	NA
Poverty income ratio	2.31 ± 1.46	2.10 ± 1.44	<.001
Duration of diabetes	10.73 ± 10.37	16.65 ± 12.15	<.001
Smoked at least 100 cigarettes in life (%)			.668
Yes	51.08	52.06	NA
No	48.92	47.94	NA
Hypertension (%)			<.001
Yes	69.60	75.48	NA
No	30.40	24.52	NA
Body mass index (kg/m^2^)	32.58 ± 7.43	32.91 ± 7.99	.737
Total cholesterol (mmol/L, mean ± SD)	4.68 ± 1.16	4.64 ± 1.18	.258
Triglyceride (mmol/L, mean ± SD)	1.60 ± 1.31	1.59 ± 1.24	.693
HDL (mmol/L, mean ± SD)	1.24 ± 0.35	1.24 ± 0.36	.747
LDL (mmol/L, mean ± SD)	2.53 ± 0.63	2.52 ± 0.65	.422
Dietary niacin (10 mg, mean ± SD)	2.25 ± 1.04	2.16 ± 1.04	.011

Mean ± SD for continuous variables: the *P* value was calculated by the linear regression model.

(%) for categorical variables: the *P* value was calculated by the chi-square test.

DR = diabetic retinopathy, HDL = high-density lipoprotein, LDL = low-density lipoprotein.

### 3.2. Relationship between dietary niacin and DR

**Figure 2.**
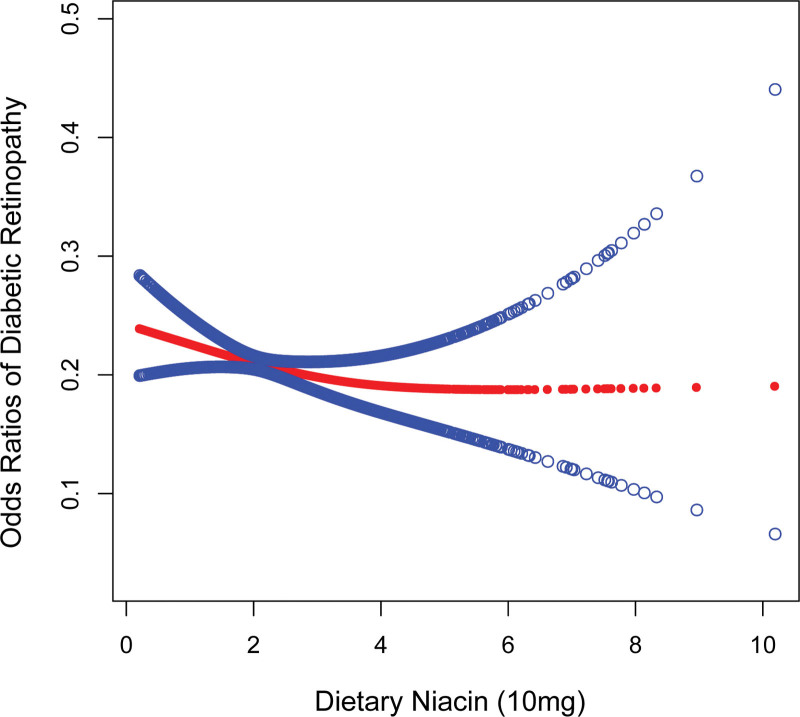
The association between dietary niacin and DR.

The negative association between dietary niacin and DR is shown in Table 2 and Figure 2. This negative correlation was significant in both the unadjusted and partially adjusted models. Dietary niacin intake was converted to a categorical variable (quartiles) during the study to explore its sensitivity. The research uncovered that individuals in the top quartile (Q4) experienced a substantial 31% decrease in the likelihood of developing DR when compared to those in the bottom quartile (Q1) of niacin consumption (OR = 0.69, 95% CI: 0.56, 0.86, *P* < .0009). Individuals in the top quartile (Q4) exhibited a 28% decreased prevalence of DR in model 3 (OR = 0.72, 95% CI: 0.57, 0.91, *P* = .0071).

**Table 2:** Relationship between dietary niacin and DR.

	Model 1 OR (95% CI) *P*-value	Model 2 OR (95% CI) *P*-value	Model 3 OR (95% CI) *P*-value
Dietary niacin (10 mg)	0.92 (0.86, 1.00) 0.0440	0.91 (0.84, 0.99) 0.0271	0.94 (0.86, 1.02) 0.1580
Q1	Reference	Reference	Reference
Q2	0.76 (0.62, 0.95) 0.0134	0.76 (0.61, 0.94) 0.0117	0.77 (0.61, 0.96) 0.0183
Q3	0.85 (0.69, 1.05) 0.1249	0.82 (0.66, 1.02) 0.0760	0.84 (0.67, 1.05) 0.1313
Q4	0.69 (0.56, 0.86) 0.0009	0.66 (0.52, 0.83) 0.0004	0.72 (0.57, 0.91) 0.0071
*P* for trend	0.86 (0.78, 0.95) 0.0037	0.84 (0.76, 0.94) 0.0015	0.88 (0.79, 0.98) 0.0212

Model 1: no covariates were adjusted. Model 2: age, gender, and race were adjusted. Model 3: age, gender, race, educational level, BMI, poverty income ratio, hypertension status, smoking status, Duration of diabetes,HDL, LDL, triglyceride, and total cholesterol were adjusted.

DR = diabetic retinopathy.

### 3.3. Subgroup analysis

To ascertain the consistency of the negative correlation between dietary niacin and DR across different populations, subgroup analyses were conducted (Table 3). The negative correlation between dietary niacin and DR remains unaffected by subgroup analyses based on age, gender, and smoking status (interaction > 0.05).

**Table 3:** Subgroup analysis for the association between dietary niacin and DR.

Characteristics	Dierary niacin intake, 10 mg				*P* for interaction
	Q1 (0.18865–1.5097) OR (95%CI)	Q2 (1.5101–2.0588) OR (95%CI)	Q3 (2.05925–2.71955) OR (95%CI)	Q4 (2.7211–10.1695) OR (95%CI)	
Gender	NA	NA	NA	NA	.7022
Male	1.00 (Ref.)	0.78 (0.55, 1.10)	0.78 (0.56, 1.08)	0.64 (0.46, 0.90)	NA
Female	1.00 (Ref.)	0.74 (0.55, 1.00)	0.89 (0.65, 1.21)	0.82 (0.56, 1.21)	NA
Age (yr)	NA	NA	NA	NA	.4988
Age < 60	1.00 (Ref.)	0.79 (0.53, 1.19)	0.75 (0.50, 1.12)	0.66 (0.44, 0.99)	NA
Age≥60	1.00 (Ref.)	0.77 (0.59, 1.01)	0.92 (0.70, 1.21)	0.78 (0.57, 1.05)	NA
Smoke	NA	NA	NA	NA	.4544
Yes	1.00 (Ref.)	0.69 (0.50, 0.94)	0.63 (0.46, 0.88)	0.64 (0.46, 0.89)	NA
No	1.00 (Ref.)	0.83 (0.61, 1.14)	1.08 (0.79, 1.47)	0.76 (0.53, 1.09)	NA

DR = diabetic retinopathy.

### 3.4. Nonlinear relationship between dietary niacin intake and DR in male

**Figure 3.**
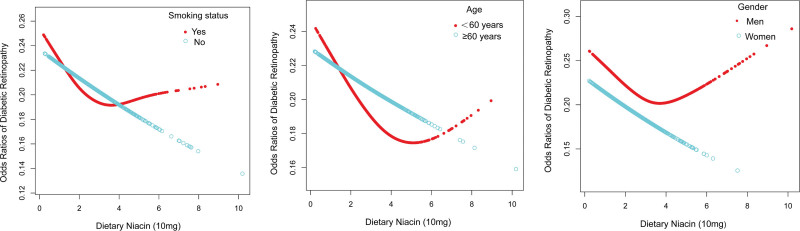
Relationship between dietary niacin and DR stratified by gende, age, and smoking status.

Smoothed curve fitting and generalized summation models were used to investigate the nonlinear relationship between dietary niacin and DR. A 2-stage linear regression model with a predicted inflection point of 3.35 (10 mg) for males revealed a nonlinear association between dietary niacin intake and DR in males (Fig. 3). To the left of the inflection point dietary niacin was negatively associated with DR (OR = 0.81, 95% CI: 0.69, 0.95, *P* = .0112), and to the right of the inflection point no statistically significant relationship was observed (OR = 1.18, 95% CI: 0.97, 1.44, *P* = .1031) (Table 4). In the nonlinear associations grouped by age and smoking status, U-shaped associations were also found for those younger than 60 years and smokers, with inflection points of 4.35 (10 mg) and 2.21 (10 mg), respectively, but none of the log-likelihood ratios were significant (Table 4).

**Table 4:** Threshold effect analysis of dietary niacin on DR using the 2-piecewise linear regression model.

Odds ratios of diabetic retinopathy	Adjusted OR (95% CI) *P*-value
Men	
Inflection point	3.35
Dietary niacin < 3.35 (10 mg)	0.81 (0.69, 0.95) 0.0112
Dietary niacin > 3.35 (10 mg)	1.18 (0.97, 1.44) 0.1031
Log-likelihood ratio	0.017
<60 years	
Inflection point	4.35
Dietary niacin < 4.35 (10 mg)	0.83 (0.71, 0.98) 0.0286
Dietary niacin > 4.35 (10 mg)	1.27 (0.89, 1.81) 0.1924
Log-likelihood ratio	0.071
Smoke	
Inflection point	2.21
Dietary niacin < 2.21 (10 mg)	0.71 (0.54, 0.93) 0.0135
Dietary niacin > 2.21 (10 mg)	1.02 (0.87, 1.20) 0.8326
Log-likelihood ratio	0.054

Age, gender, race, educational level, BMI, poverty income ratio, hypertension status, smoking status, duration of diabetes, HDL, LDL, triglyceride, and total cholesterol were adjusted.

DR = diabetic retinopathy.

## 4. Discussion

In this cross-sectional study, which encompassed a total of 3990 participants, it was discovered that greater consumption of niacin in one’s diet led to a decreased likelihood of developing DR. And this association remained stable across subgroups. A nonlinear association was detected in male subjects with an inflection point of 3.35 (10 mg). The findings suggest that increasing niacin intake in the daily diet may reduce the prevalence of DR.

To the best of our understanding, this represents the initial cross-sectional examination that evaluates the correlation between dietary niacin intake DR. According to reports, niacin has previously been explored in various fields, particularly endocrine diseases (e.g., diabetes) and cardiovascular diseases.^[16–19]^ A cross-sectional study involving 3106 subjects from China found a negative association between dietary niacin intake and the prevalence of diabetes, conducted by Jiang et al.^[20]^ After accounting for all relevant factors, an increase of 1 unit in the intake of dietary niacin was found to be linked with a noteworthy 3.5% decrease in the likelihood of developing diabetes[OR = O.965, 95% CI: (0.944, 0.986), *P* = .001]. In another study, Liu et al used data from NHANES 2005–2016, which included a total of 24,494 subjects who met the criteria.^[18]^ The study arrived at a comparable finding, showing a clear and noteworthy link between a higher intake of niacin in one’s diet and a reduced likelihood of developing diabetes. However, some studies have reached different conclusions. According to the study conducted by Pan et al, it was discovered that a considerable intake of niacin in one’s diet had a positive correlation with the occurrence of diabetes among adults in the United States.^[19]^

Despite not fully comprehending the physiological processes that account for the negative relationship between niacin consumption and DR, we can shed light on this matter through various potential pathways. First, in the pathogenesis of DR, the impaired mitochondrial membrane potential in the retina produces leakage and swelling, leading to mitochondrial dysfunction that affects the electron transport chain and impedes ATP production.^[21,22]^ Niacin, better known as vitamin B3, is a water-soluble vitamin that serves as the nutritional precursor of NAD and NADP.^[8]^ According to studies, niacin administration raises NAD levels in cells and mice, which activates sirtuins.^[23–25]^ Sirtuins (SIRT1 and SIRT3) have been reported to improve mitochondrial biogenesis and function, and their overexpression enhances mitochondrial metabolic health.^[26–28]^ On the other hand, oxidative stress is a significant factor in the development of DR. Excessive reactive oxygen species can damage retinal blood vessels and their surrounding tissues, leading to the development of DR.^[29]^ Specifically, NADPH oxidase (NOX), a key zymogen of oxidative stress, is capable of converting molecular oxygen into peroxides.^[30]^ The NOX system comprises multiple subgroups, and it has been found that when retinal cells are exposed to high glucose levels, NOX 2 is strongly activated. This enhances the expression of intercellular adhesion factor-1 and vascular endothelial growth factor, which further promotes neovascularization and induces more severe DR.^[31–33]^ Prior studies have evidenced the capacity of niacin to alleviate oxidative stress in endothelial cells. This is accomplished by lowering NOX levels, which in turn inhibits the formation of reactive oxygen species.^[34]^

Beyond these mechanistic considerations, our subgroup analysis also revealed nonlinear associations among male subjects. This may be related to gender differences in dietary niacin absorption and metabolism. Previous studies have shown that at certain doses, plasma niacin levels peak more rapidly in male populations.^[35]^ This implies earlier niacin exposure in male bloodstreams, potentially leading to quicker saturation. Excessive niacin intake can adversely affect glucose metabolism.^[36]^ Female estrogen plays a crucial role in regulating metabolism and anti-inflammatory effects, providing female with additional protection.^[37,38]^ Concurrently, higher smoking and drinking rates among male contribute to these factors, collectively leading to differences in niacin’s protective effects across genders.^[39,40]^

According to the Dietary Reference Intakes established by the Institute of Medicine, the Dietary Reference Intake for niacin ranges from 6 to 16 mg/d across different age groups, with a tolerable upper intake level (UL) of 35 mg/d for dietary niacin. Detailed information can be obtained by querying https://ods.od.nih.gov/factsheets/Niacin-HealthProfessional/. In our study, men exhibited a nonlinear relationship with a turning point around 3.35 (10 mg), a value extremely close to the established UL. This suggests that moderate niacin intake may help reduce the prevalence of DR, but intake levels approaching the UL may not provide additional protective benefits and should be approached with caution. Previous studies have also highlighted the potential risks of niacin intake exceeding the UL for DR and other ocular diseases, such as retinal vein occlusion and glaucoma.^[41,42]^ It is particularly important to note that although UL is primarily established based on adverse reactions associated with pharmacological doses or supplemental forms of niacin (rather than natural food sources), this study also emphasizes that assessing potential risks is critically important when dietary niacin intake approaches the threshold for adverse reactions.

The utilization of a database that is typical of the U.S. population provided the study’s data with more reliability and representativeness, which is its main strength. Secondly, the sample size was large enough to allow subgroup analysis of people of different ages, gender, and BMI. However, several limitations of this study warrant clarification. First, despite adjusting for multiple potential confounders, residual confounding effects from other lifestyle or dietary factors cannot be entirely ruled out. Second, the clinical applicability of these findings remains unclear. Although niacin is a known nutritional supplement and essential nutrient, this study relied on dietary intake data, leaving the optimal dosage and differences in benefits between dietary sources and supplements undefined. Third, a gender-specific nonlinear effect was observed in the male cohort, but the potential mechanisms underlying this difference require further clarification. Fourth, participants’ dietary intakes were assessed via dietary recall interviews, potentially subject to memory bias and classification errors. Finally, the cross-sectional study design precludes causal inference. Longitudinal or interventional studies are needed to validate these findings and establish optimal niacin intake levels for preventing DR.

## 5. Conclusion

In summary, this study reveals a negative correlation between dietary niacin intake and DR, providing preliminary evidence for incorporating dietary niacin into nutritional strategies for preventing DR. From a public health perspective, dietary niacin is inexpensive and readily accessible. Moderately increasing niacin intake through daily dietary management may serve as a feasible strategy for eye health.

## Author contributions

**Conceptualization:** Meirong Chen.

**Data curation:** Shilong Wang, Xiao Li.

**Formal analysis:** Shilong Wang.

**Investigation:** Shilong Wang.

**Methodology:** Shilong Wang.

**Resources:** Shilong Wang, Xiao Li.

**Software:** Shilong Wang, Xiao Li.

**Writing – original draft:** Shilong Wang, Xiao Li.

**Writing – review & editing:** Shilong Wang, Xiao Li, Meirong Chen.
